# Supplementary material for: Comparative Analysis Reveals Host Species-Dependent Diversity Among 16 Virulent Bacteriophages Isolated Against Soybean Bradyrhizobium spp
Source: Viruses. 2025 Nov 4;17(11):1474. doi: 10.3390/v17111474 (PMC12656738; doi:10.3390/v17111474)
Supplement: Supplementary file 1 [file viruses-17-01474-s001.zip › Revised supplementary tables and figures by Morgese et al. (viruses-3934817) - (CLEAN) copy.pdf]

Supplementary Materials

Table S1. Mean measurements of morphological characteristics for 16 virulent *Bradyrhizobium* phages isolated from Delaware soils. Individual phages were measured for capsid diameter, capsid length, tail diameter, and tail length. Capsid volumes were calculated using a formula for the volume of an ellipsoid.

Isolation host species	Phage isolate	Sample size (n)	Capsid diameter (nm)	Capsid length (nm)	Capsid volume (10^5 nm^3)	Tail diameter (nm)	Tail length[2] (nm)
B. elkanii	A31	15	57.4	71.9	1.04	12.7	126.5
	E31	15	57.3	70.8	1.02	12.9	128.9
	F31	10	56.6	67.7	0.96	13.6	132.5
	H31	22	59.0	72.4	1.04	12.5	125.3
	A94	16	61.3	70.4	1.25	12.7	118.8
	B94	18	59.1	70.8	1.08	13.5	126.2
	E94	15	59.9	72.3	1.13	13.7	127.7
	G94	16	58.7	69.7	1.06	13.2	134.4
	H94	15	60.3	75.7	1.15	11.5	132.1
	J94	15	60.4	74.0	1.16	11.9	125.7
	K94	23	57.0	72.8	1.11	11.4	126.1
	L94	17	58.2	71.4	1.04	11.0	123.3
B. diazoefficiens	D110	31	73.1	70.9	1.88	nd[1]	16.5
	F110	31	67.8	66.9	1.65	nd	16.6
	L110	31	65.1	66.3	1.46	nd	16.9
	J110	16	59.6	66.8	1.12	nd	16.2

[1]nd, not determined. Phages isolated against USDA 110 displayed a podophage-like morphology that prevented reliable tail diameter measurements.

[2]Tail length of *B. diazoefficiens* phages is not representative of the full sample size due to positionally obscured tails for some images. Sample sizes for tail measurements are as follows: D110: n=22, F110: n=14, L110: n=12, J110: n=6.

Table S2. Genomic features of 16 virulent *Bradyrhizobium* phages isolated from Delaware soils. Genome length in base pairs, percent GC content, total number of genes, and the number of functionally annotated genes are shown.

Isolation host species	Lytic phage isolate	Total genome length (bp)	GC content (%)	Total number of genes[1]	Genes with functional annotations[1] (%)
	A31	63,461	67.0	101	24.75
	E31	62,868	67.1	99	25.25
	F31	63,113	67.1	101	24.75
	H31	63,205	66.9	102	24.51
	A94	62,724	67.0	99	25.25
B. elkanii	B94	62,580	67.1	99	25.25
	E94	62,507	67.1	99	25.25
	G94	63,251	66.9	100	25.00
	H94	63,205	67.0	100	25.00
	J94	64,087	66.7	103	24.27
	K94	62,466	66.9	99	24.24
	L94	63,206	67.0	97	25.77
	D110[2]	57,535	43.0	105	21.90
B. diazoefficiens	F110[3]	55,636	43.5	98	20.41
	L110[2]	57,005	44.2	105	21.90
	J110	41,973	58.9	78	19.23

[1]Includes tRNA and tmRNA genes

[2]Genome contains tRNAs: tRNA-Leu (taa), tRNA-Thr (tgt)

[3]Genome contains tRNAs: tRNA-Leu (taa), tRNA-Thr (tgt), and tRNA-Met (cat)

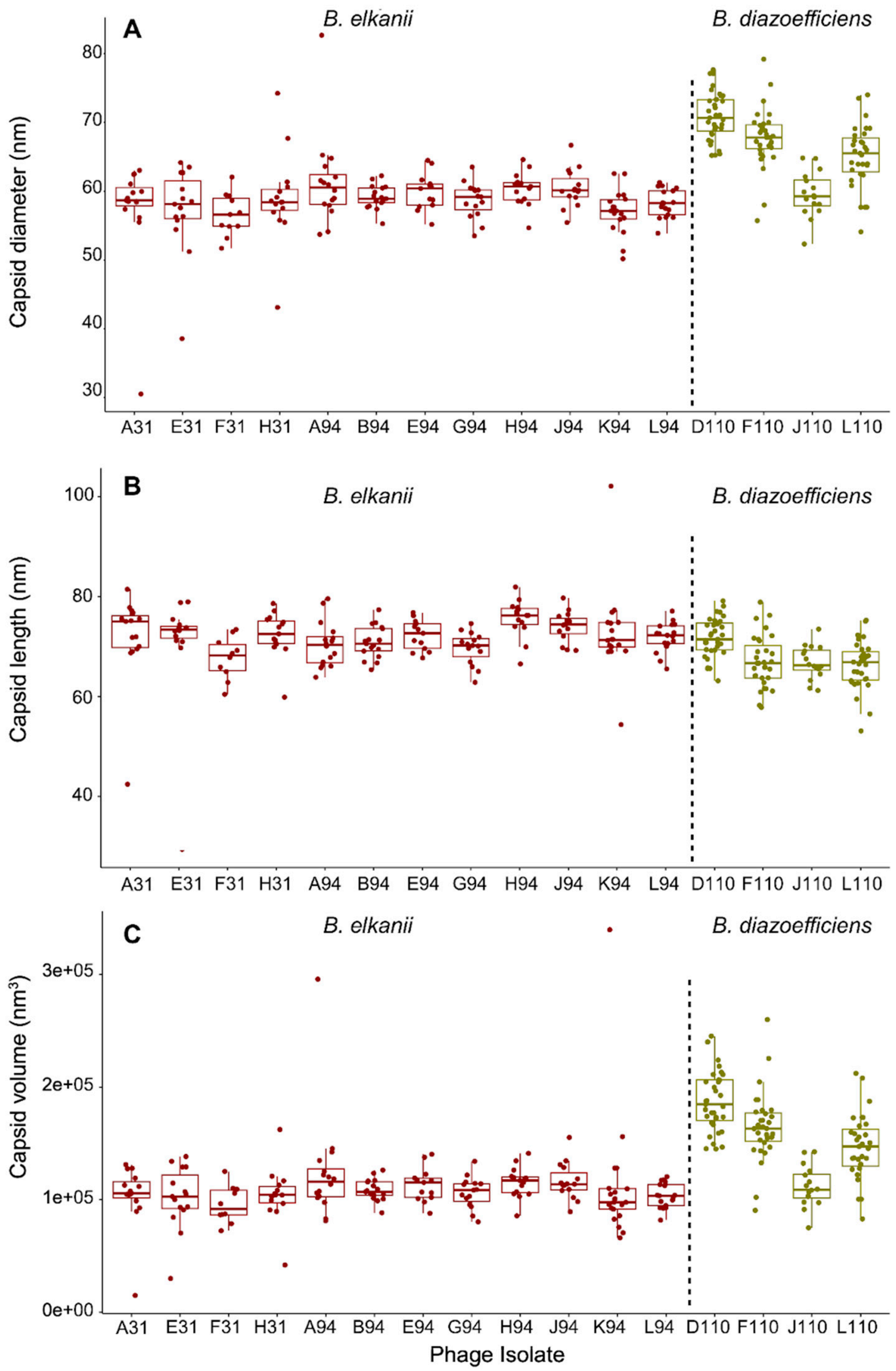

Figure S1. Boxplots of capsid dimensions and estimated capsid volumes of the 16 virulent bacteriophages isolated against *Bradyrhizobium elkanii* and *B. diazoefficiens*. Phage isolates are listed on the x-axis, with the capsid dimensions and volumes shown on the y-axis: (A) capsid diameter, (B) capsid length, and (C) capsid volume. Individual box plots and data points are colored according to the species of *Bradyrhizobium* used for isolation: *B. elkanii*, red; *B. diazoefficien*s, green.

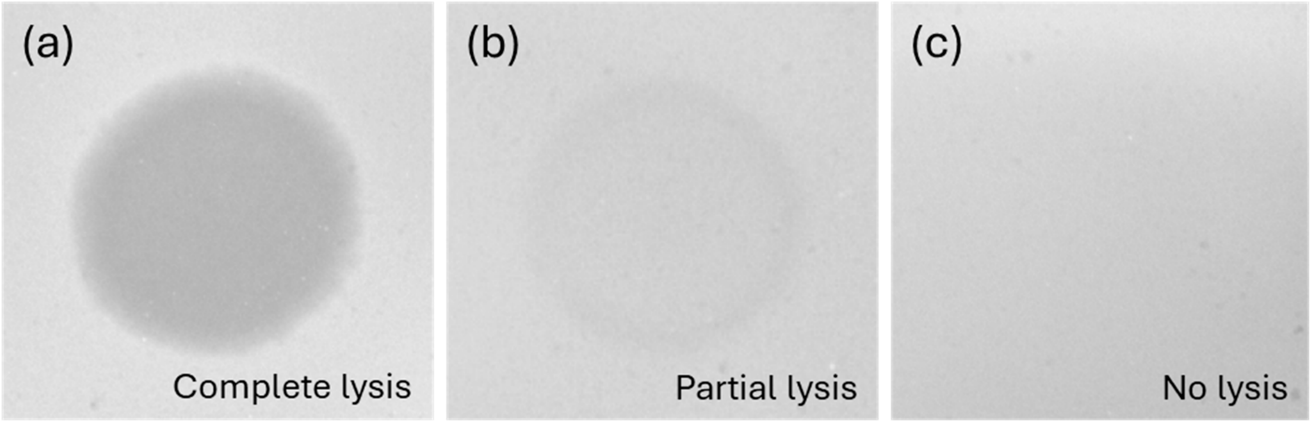

Figure S2. Representative host range spot assay results showing the varying levels of lytic activity by *Bradyrhizobium* phages. (a) Complete lysis: clear zone (b) Partial lysis: turbid zone (c) No lysis: absence of clearing.

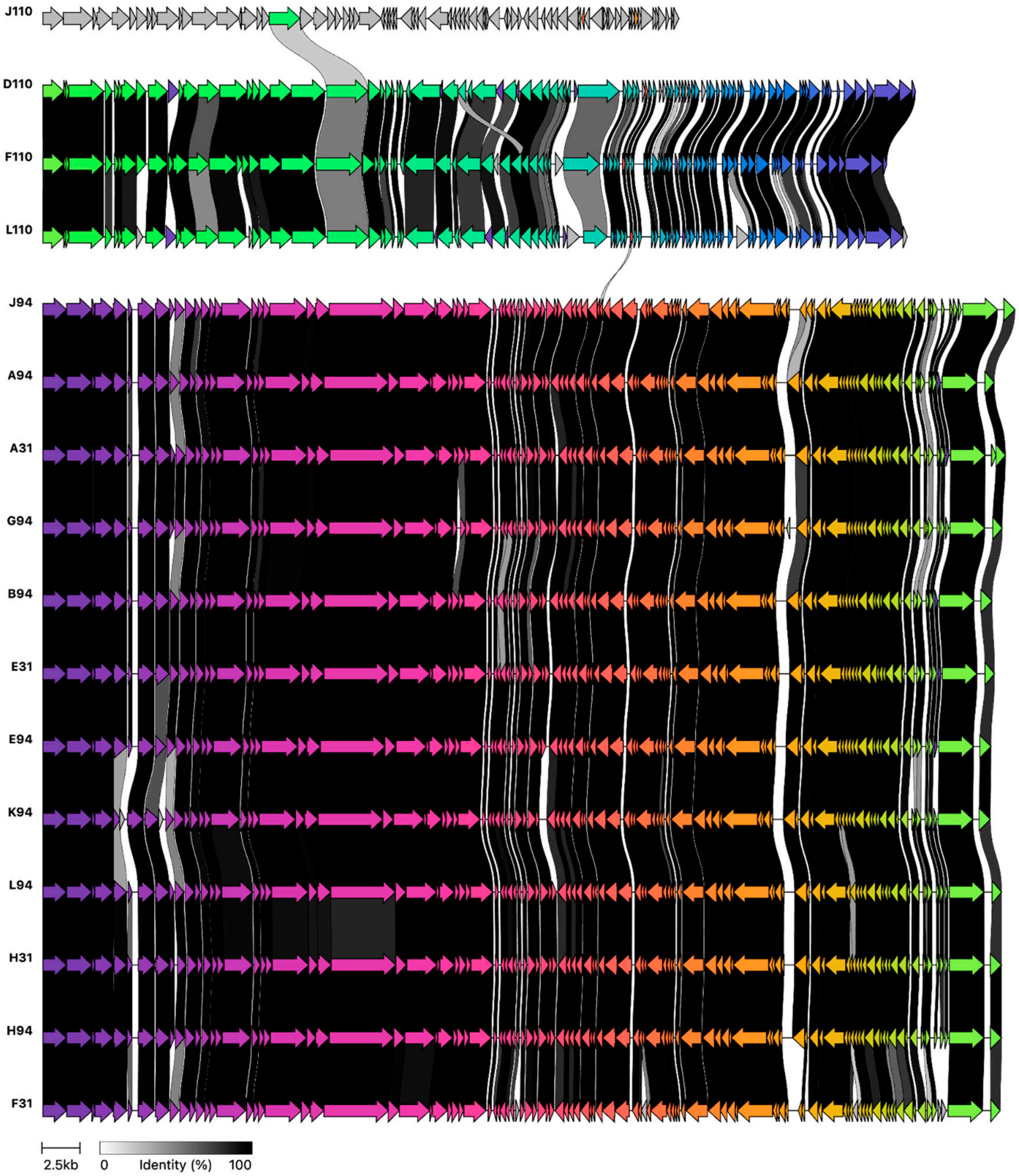

Figure S3. Clinker alignment of the complete genomes of 16 lytic *Bradyrhizobium* phages isolated from Delaware soils, showing protein and nucleotide level similarity consistent with ANI-based species groupings. Each row represents a phage genome, oriented from the large subunit terminase gene. Arrows denote individual genes, scaled by length and colored by Clinker-defined protein clusters; direction indicates strand orientation. Links between adjacent genomes represent nucleotide-level identity, shaded by percent identity.

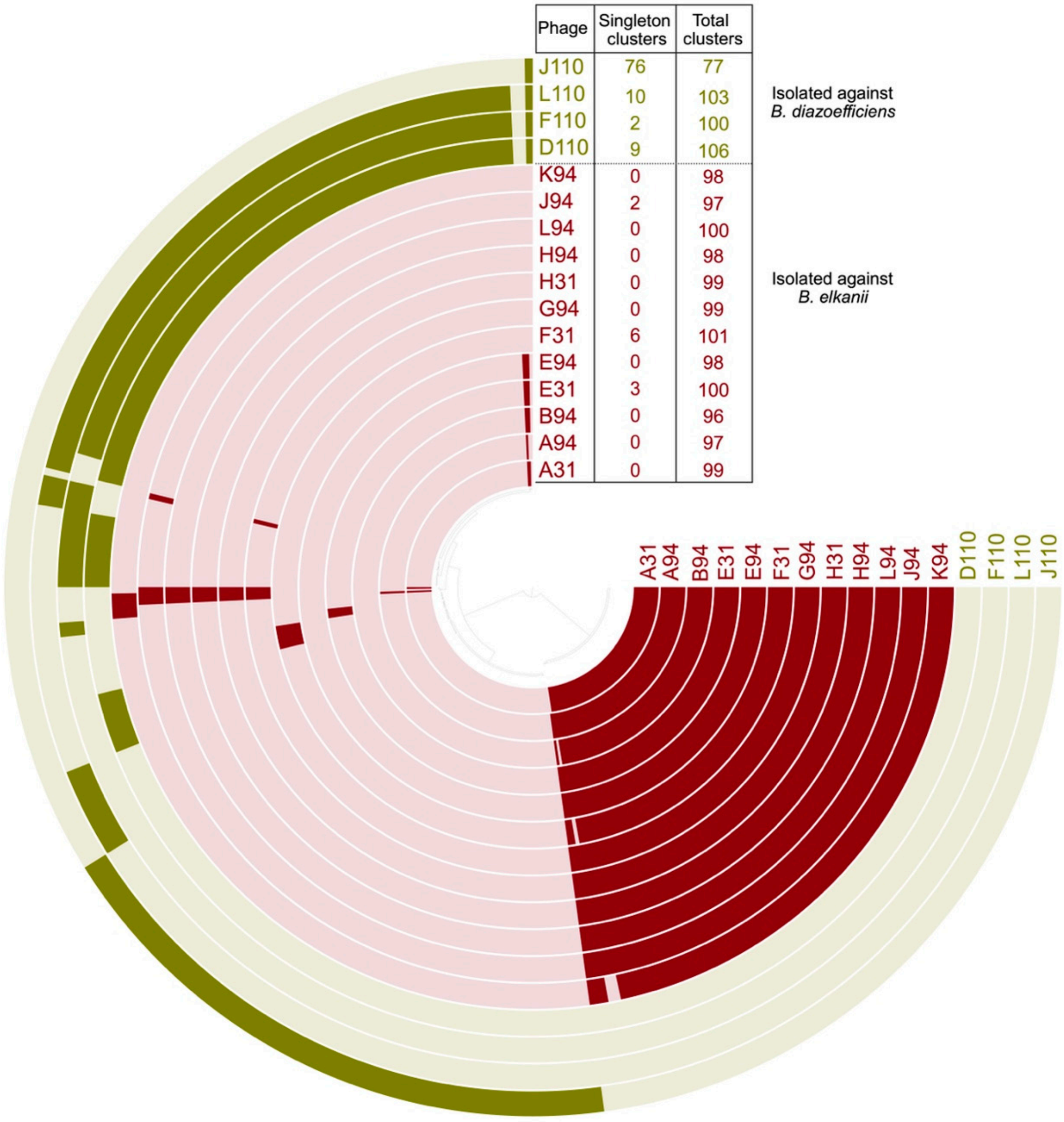

Figure S4. Pangenome display of 16 phages virulent on soybean *Bradyrhizobium* spp. isolated from Delaware soils. The pangenome clusters based on presence/absence across the 16 genomes (Euclidean distance; Ward linkage). The center tree arranges the gene clusters identified across the genomes. Each concentric layer represents a phage genome, with dark shading indicating the presence of a gene cluster in that genome, and light shading representing the absence of a gene cluster. The positioning of the gene clusters does not represent their order in the genomes. Layers are grouped and color-coded by the original isolation host species: *B. elkanii* (red), *B. diazoefficiens* (green). This figure highlights the core, accessory, and unique gene clusters, showing both the conservation and variability across the three phage populations.
